# Polyphosphate Activates von Willebrand Factor Interaction with Glycoprotein Ib in the Absence of Factor VIII In Vitro

**DOI:** 10.3390/ijms232214118

**Published:** 2022-11-15

**Authors:** Marcela Montilla, Isabel Atienza-Navarro, Francisco Jose García-Cozar, Carmen Castro, Francisco Javier Rodríguez-Martorell, Felix A. Ruiz

**Affiliations:** 1 Research Unit, Hospital Universitario Puerta del Mar, 11009 Cadiz, Spain; 2 Instituto de Investigación e Innovación Biomédica de Cádiz, 11009 Cadiz, Spain; 3 Medical School, Universidad Cooperativa de Colombia, Villavicencio 500003, Colombia; 4 Department of Biomedicine, Biotechnology, and Public Health, Medical School, Universidad de Cádiz, 11003 Cadiz, Spain; 5 Department of Hematology, Hospital Universitario Virgen del Rocío, 41013 Seville, Spain

**Keywords:** factor VIII, hemophilia A, polyphosphates, von Willebrand diseases, von Willebrand factor

## Abstract

Polyphosphate (polyP), a phosphate polymer released by activated platelets, may modulate various stages of hemostasis by binding to blood proteins. In this context, we previously reported that polyP binds to the von Willebrand factor (VWF). One of the most significant functions of VWF is to bind to and protect the blood circulating Factor VIII (FVIII). Therefore, here, we study the role of polyP in the VWF–FVIII complex in vitro and suggest its biological significance. Surface plasmon resonance and electrophoretic mobility assays indicated that polyP binds dynamically to VWF only in the absence of FVIII. Using the VWF Ristocetin Cofactor assay, the most accepted method for studying VWF in platelet adhesion, we found that polyP activates this role of VWF only at low levels of FVIII, such as in plasmas with chemically depleted FVIII and plasmas from severe hemophilia A patients. Moreover, we demonstrated that FVIII competes with polyP in the activation of VWF. Finally, polyP also increases the binding of VWF to platelets in samples from patients with type 2 and type 3 von Willebrand disease. We propose that polyP may be used in designing new therapies to activate VWF when FVIII cannot be used.

## 1. Introduction

Inorganic polyphosphates (polyPs) are natural linear polymers of phosphate secreted by activated platelets [1] and mast cells [2], recently recognized as potent modulators of hemostasis, thrombosis, and inflammation [3].

The most studied functions of polyP in the blood are concerned with the modulation of clotting factors at different levels, including the activation of factor XII [4], factor XI [5], factor V [6], and thrombin [7] and the inactivation of tissue factor pathway inhibitor [8]. PolyP also inhibits the fibrinolytic pathway [4,9], activates the complement system [10,11], and stabilizes fibrin clotting [9,12].

We recently demonstrated that polyP may also contribute to primary hemostasis, promoting the biological activity of the von Willebrand Factor (VWF) [13]. The VWF is a multimeric glycoprotein that mediates platelet adhesion to damaged vascular subendothelium through its interactions with platelet glycoprotein receptor Ib (GP1b) and subendothelial collagen (reviewed in [14,15]). We described that polyP promotes the interaction of VWF with platelet GP1b [13].

Other activities of VWF, such as its binding to collagen, were unaffected by polyP [13]. The multimerization state of VWF, which is decisive for some of its adhesion activities [16,17], also did not change in VWF incubated with polyP or polyP-degrading enzymes [13].

VWF is present in plasma and accumulates in the platelet α-granules and Weibel–Palade bodies of endothelial cells [18]. We found polyP in fractions of VWF isolated from normal human platelets and plasma, thus demonstrating that this protein normally circulates with polyP bound to it [13].

One of the important functions of VWF is the stabilization of circulating factor VIII (FVIII), a co-factor in the intrinsic tenase complex [19,20]. This is achieved by the formation of a tight, non-covalently linked VWF–FVIII complex, which protects FVIII from degradation by activated C protein [18].

Quantitative or functional defects of VWF are known as von Willebrand disease (VWD), which is the most common inherited bleeding disorder in humans [21]. VWD is classified into three main types: partial quantitative deficiency (type 1), qualitative deficiency (type 2), and complete quantitative deficiency (type 3). Type 2 VWD is further divided into four variants (2A, 2B, 2N, 2M) based on the characteristics of the dysfunctional VWF [17,22]. In the majority of the cases of VWD, as VWF is absent and/or unavailable to bind and protect FVIII, the levels of both proteins fall in parallel [15].

A congenital deficiency in FVIII results in hemophilia A, a bleeding disorder that is currently treated with protein replacement therapy using either plasma-derived or recombinant FVIII [20]. In hemophilia A, the amount of circulating VWF is similar to that in healthy individuals; therefore, VWF and FVIII levels are used in the differential diagnosis of the disease [15].

In this study, we investigated the role of polyP in the VWF–FVIII complex using plasmas from patients with hemophilia A, samples in which FVIII was chemically depleted, and plasmas from patients with different types of VWD. The results obtained demonstrate that FVIII competes with polyP in the activation of VWF binding to GP1b.

## 2. Results

### 2.1. Effect of Factor VIII on the Interaction between VWF and PolyP

We previously described that polyP binds to VWF in vitro and in vivo [13]. Therefore, we analyzed the effect of Factor VIII on the molecular interaction between VWF and polyP.

**Figure 1:**
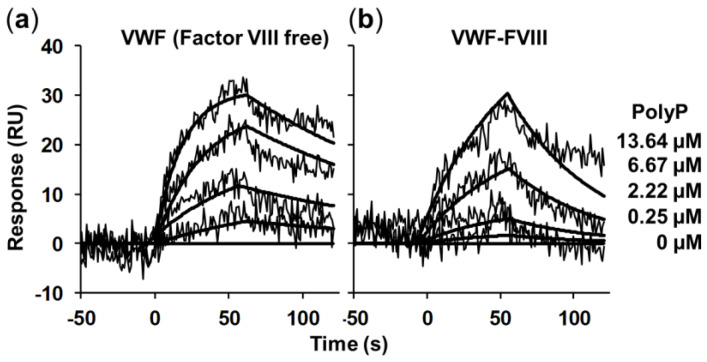
Dynamic interaction between von Willebrand Factor and polyphosphate is stronger in the absence of factor VIII. Surface plasmon resonance analysis of immobilized von Willebrand Factor (factor VIII-free) (VWF) (**a**) or von Willebrand factor co-purified with factor VIII (VWFFVIII) (**b**), confronted with increasing concentrations of isolated polyphosphate (polyP_65_, 0.25–13.34 µM). The corresponding fitted curves are presented as solid lines. A representative experiment is shown (n = 3).

Here, we first studied real-time interactions between polyP and VWF using surface plasmon resonance (Figure 1) in the presence and absence of factor VIII. The affinity between polyP_65_ and protein decreased drastically when we used a VWF co-purified with factor VIII (Figure 1B). The calculated K*ds* for polyP_65_ (in terms of its phosphate residues) were: 1.82 ± 0.25 µM to VWF (factor VIII free) and 0.29 ± 0.04 M to VWF isolated with factor VIII (10^5^ times less affinity when factor VIII was present).

**Figure 2:**
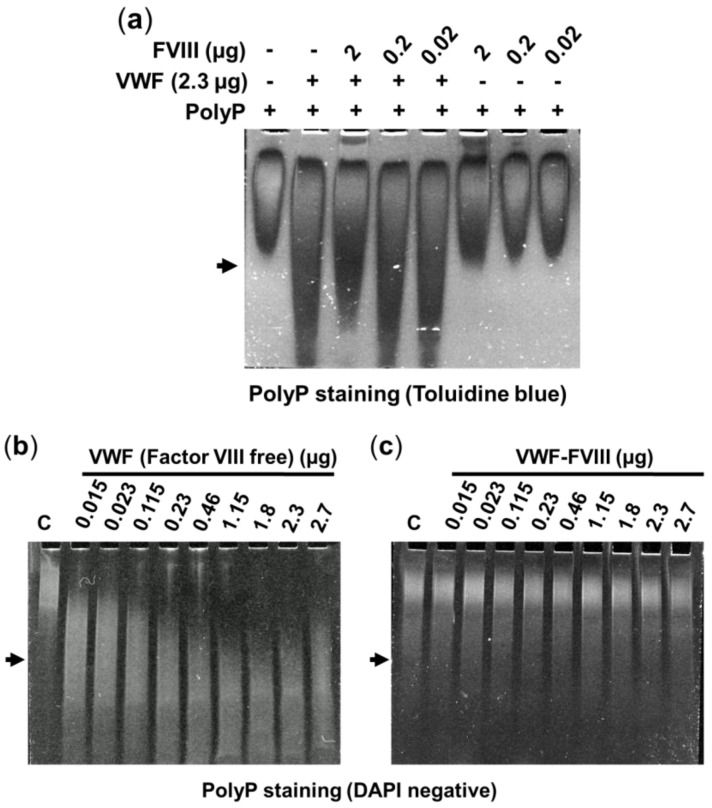
Characterization of the polyphosphate-von Willebrand Factor binding, in the absence or presence of factor VIII, using urea–polyacrylamide gel electrophoresis. (**a**) Purified von Willebrand factor (factor VIII-free, 2.3 µg) and/or purified factor VIII (0.02 to 2 µg) were incubated with 0.8 mM of isolated polyphosphate (polyP_65_). Samples were loaded onto urea–polyacrylamide gels, separated by electrophoresis, and stained using a polyphosphate-specific staining (toluidine blue). Arrows show the mobility of von Willebrand factor on the gels. Representative experiments are shown (n = 3). (**b**,**c**) Increasing amounts of von Willebrand factor, either factor VIII-free in (**b**), or von Willebrand factor co-purified with factor VIII in (**c**), were incubated with 0.8 mM of isolated polyphosphate (polyP_65_) as described in Methods. Samples were separated as before and stained using a sensitive polyphosphate-specific staining (DAPI negative). Similar experiments, but stained with toluidine blue, are shown in Supplementary Figure S1.

Similar results were observed using urea-PAGE (Figure 2 and Figure S1). As we previously demonstrated, the electrophoretic mobility of polyP_65_ in urea–PAGE could be modified by the addition of isolated VWF, which migrates faster than free polyP [13]. The inclusion of purified factor VIII prevented changes in the electrophoretic mobility of polyP_65_ in a dose-dependent manner (Figure 2a). When we used samples of VWF that contained endogenous factor VIII (VWF co-purified with factor VIII), binding with polyP was not detected (Figure 2c and Figure S1b).

### 2.2. PolyP Increases VWF Ristocetin Co-factor Activity in Factor VIII-Deficient Plasmas

**Figure 3:**
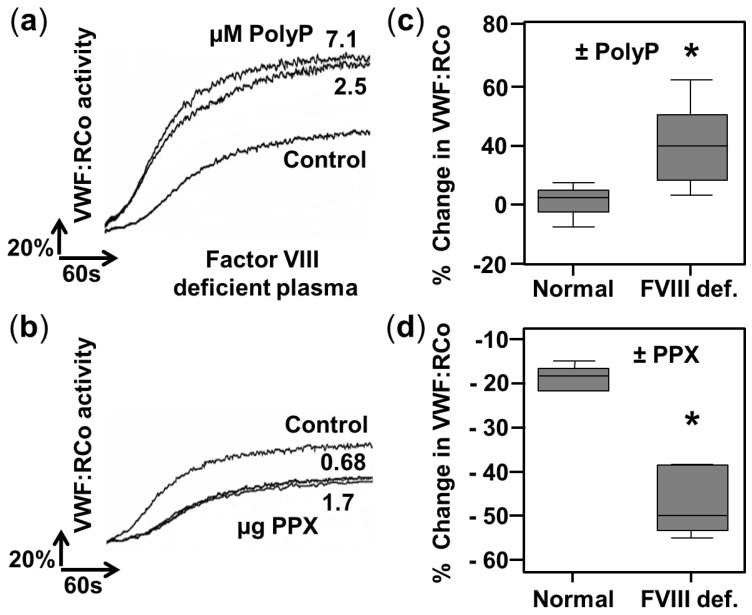
PolyP increases the von Willebrand Factor ristocetin cofactor activity in factor VIII-deficient plasmas. (**a**,**b**) Aggregation curves of fixed platelets in a ristocetin co-factor activity assay using factor VIII-deficient plasmas. Prior to the assay, 50 µL of plasmas (diluted 1:2) were incubated in the absence (control) or in the presence of (**a**) isolated polyphosphate (polyP_65_, 2.5 µM, and 7.1µM) and (**b**) recombinant yeast exopolyphosphatase (PPX, 0.68 µg, and 1.7 µg). Representative experiments are shown (n = 3). (**c**,**d**) Quantification of the differences in von Willebrand factor ristocetin co-factor activity in normal plasmas (Normal) and plasmas depleted of factor VIII (FVIII-def.) after the addition of (**c**) 2.5 µM of isolated polyphosphate (polyP_65_) or (**d**) 1.7µg of recombinant yeast exopolyphosphatase (PPX). For each sample, the change was calculated as “activity without addition of polyP or PPX” minus “activity with the addition of polyP or PPX”; therefore, positive values mean an increase above the basal activity and negative values mean a decrease. Results are presented in a box-and-whiskers plot. An asterisk indicates a statistical difference of *p* < 0.01, determined by Mann–Whitney test using plasmas of 3 normal individuals vs. factor VIII-depleted plasmas from three different lots. Measurements were performed using an automated aggregometer (Helena AggRAM).

In a previous work, we demonstrated that the addition of polyP produces an increase in von Willebrand factor ristocetin co-factor (VWF:RCo) activity in plasmas with low VWF (from type 1 VWD patients) [13]. Here, we tested the effect of polyP concentration on plasmas in which factor VIII is chemically depleted (Figure 3).

The addition of increasing concentrations of polyP_65_ doubled the levels of VWF:RCo activity (Figure 3a), and this change is statistically significant in comparison to normal plasmas (Figure 3c). In concordance, incubation with the enzyme exopolyphosphatase (PPX), which specifically degrades polyP, decreased the VWF:RCo activity more strongly in factor VIII-deficient plasmas, in comparison with normal plasmas (Figure 3b,d).

Surprisingly, the addition of purified factor VIII also increased the VWF:RCo activity in factor VIII-deficient plasmas (Supplementary Figure S2). Purified factor VIII was a considerably more potent activator of VWF:RCo activity than polyP: it increased basal VWF:RCo activity by a factor of four (Supplementary Figure S2). However, if polyP and purified factor VIII were added together, the activation of VWF:RCo activity was slightly (but significantly) lower, suggesting a competitive effect between the two factors (Supplementary Figure S1).

In addition, we analyzed the effect of polyP of different lengths in the activation of VWF:RCo activity in Type 1 VWD and factor VIII-depleted plasmas (Supplementary Figure S3). A concentration of 2.5 µM orthophosphate and similar concentrations (expressed in phosphate residues) of pyrophosphate, tripolyphosphate, tetrapolyphosphate, and polyP_25_, polyP_45_, and polyP_65_ were tested (Supplementary Figure S3). Only polyPs with a length greater than 45 phosphate residues significantly increased the VWF:RCo activity (Supplementary Figure S3).

### 2.3. PolyP Increases VWF Ristocetin Co-factor Activity in Severe Hemophilia A Plasmas

**Figure 4:**
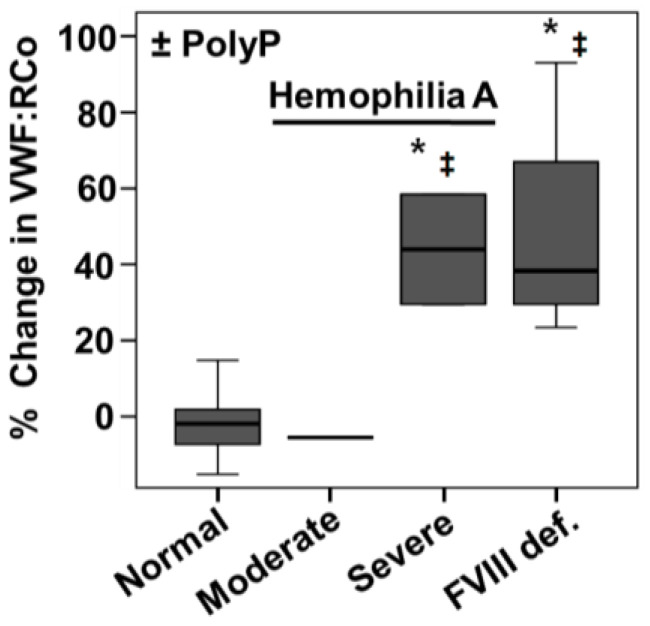
PolyP addition increases the von Willebrand factor activity in plasmas from patients with severe hemophilia A. Experiments were performed as described in Figure 3 but using plasmas from healthy individuals (Normal) and patients with moderate and severe hemophilia A (Moderate, Severe). Quantification of the differences in von Willebrand factor ristocetin co-factor activity after the addition of 2.5 µM of isolated polyphosphate (polyP_65_). For each sample, the change was calculated as “activity without addition of polyP” minus “activity with the addition of polyP”. For comparison, we also include data from experiments using factor VIII-deficient plasmas (FVIII-def.). Results are presented in a box-and-whiskers plot. Symbols indicate a statistical difference of *p* < 0.01, determined by Mann–Whitney test, related to “Normal” plasmas (*), or “Moderate hemophilia” (‡) (n = 3). An asterisk indicates a statistical difference of *p* < 0.01, determined by Mann–Whitney test. Measurements were performed using an automated aggregometer (Helena AggRAM).

We next tested the effect of polyP on plasmas from hemophilia A patients (Figure 4). In plasmas from patients with severe hemophilia A (less than 1% of normal factor VIII activity), the addition of polyP_65_ increased the VWF:RCo, similar to its effect in plasmas chemically depleted of factor VIII (Figure 4). On the contrary, in plasmas from patients with moderate hemophilia A (with 1 to 20% of factor VIII activity in the blood), the addition of polyP did not significantly change the VWF:RCo activity (Figure 4).

### 2.4. Exopolyphosphatase Cannot Inhibit VWF Ristocetin Co-factor Activity in the Presence of Factor VIII

**Figure 5:**
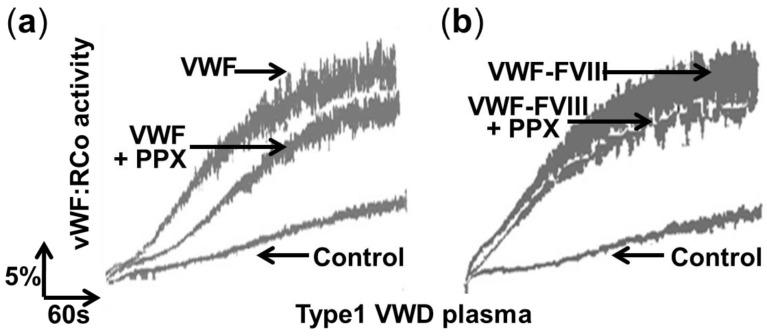
PolyP depletion does not affect von Willebrand factor ristocetin co-factor activity in the presence of factor VIII. Aggregation curves of fixed platelets in a ristocetin co-factor activity assay of plasma from patients with von Willebrand disease type 1. Prior to the assay, 50 µL of plasmas (diluted 1:2) were incubated for 10 min at 37 °C in the absence (control) or in the presence of 3.5 µg of von Willebrand factor (factor VIII-free) (VWF) (**a**) or von Willebrand factor co-purified with factor VIII (VWF–FVIII) (**b**). In addition, we performed similar plasma incubations but including 6.4 µg of recombinant yeast exopolyphosphatase (+PPX) (**a**,**b**). Measurements were performed using an optical platelet aggregometer (Chronolog Aggro-meter model 400). A representative experiment is shown (n = 3).

We also tested the effect of polyP depletion with the polyP-degrading enzyme exopolyphosphatase (PPX) in plasmas of type 1 VWD supplemented with VWF (Figure 5). The addition of VWF (factor VIII-free or co-purified with factor VIII) increased the VWF:RCo activity (Figure 5a,b). However, incubation with PPX decreased the VWF:RCo activity only when factor VIII-free VWF was used (Figure 5a).

### 2.5. PolyP Increases VWF Ristocetin Co-factor Activity on Type 2 and Type 3 VWD

**Figure 6:**
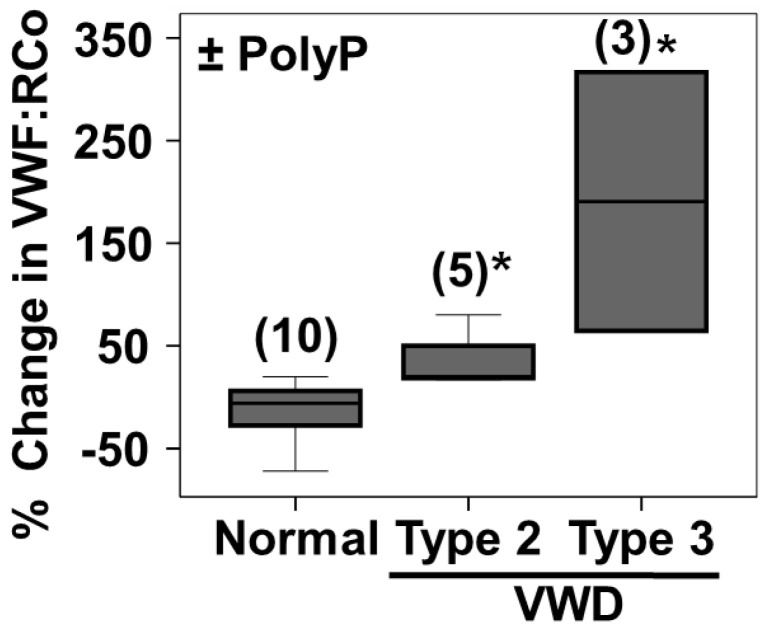
PolyP addition increases the von Willebrand factor ristocetin co-factor activity in plasmas from patients with von Willebrand disease type 2 and 3. Experiments were performed as described in Figure 3 but using plasmas from healthy individuals (Normal) and patients with von Willebrand disease type 2 (Type 2 VWD) and type 3 (Type 3 VWD). Quantification of the differences in von Willebrand factor ristocetin co-factor activity after the addition of 2.5 µM of isolated polyphosphate (polyP_65_). Results are presented in a box-and-whiskers plot. An asterisk indicates a statistical difference of *p* < 0.01, determined by Mann–Whitney test. Numbers of individuals in each category are indicated in parenthesis. Measurements were performed using an automated aggregometer (Helena AggRAM).

PolyP_65_ was also able to significantly activate the VWF:RCo activity in plasmas from patients of Type 2 and Type 3 VWD (Figure 6). The increase was greater in plasmas from Type 3 VWD patients (Figure 6).

## 3. Discussion

In this study, we demonstrated that polyP and FVIII compete in the modulation of the interaction between VWF and platelet GPIb.

**Figure 7:**
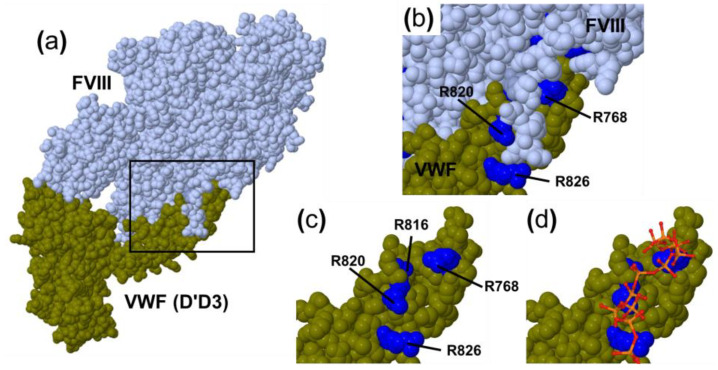
Predicted interaction of polyP with VWF. (**a**) The most recent structure of the FVIII–VWF complex (D′D3 domain), from reference [24] (PDB code 7KWO). The square indicates the area zoomed in on in the other panels of this figure. (**b**) Interaction between the FVIII-a3 acidic peptide and the VWF-highly basic groove. Arginines are labelled in blue. (**c**) VWF-highly basic groove without FVIII. (**d**) PolyP was superposed with the VWF highly basic groove. The model for polyP was extracted from the PDB (code 5LLF). Images were created using Mol* [25].

The analysis of the physical interaction between polyP and VWF showed that it only occurs in the absence of FVIII (Figure 1 and Figure 2). According to our results, FVIII may be competing with polyP, thus inhibiting its binding and the activation of VWF. Therefore, we hypothesize that the polyP-binding site of VWF overlaps with that of FVIII (Figure 7). The binding functions of VWF have been mapped to discrete domains in its monomeric structure [15]. D’-D3 domains, localized at the N-terminal of mature VWF, represent the binding site for FVIII [23]. A recent structural analysis determined that a critical determinant of the FVIII–VWF complex's formation is a FVIII acidic peptide region that inserts into a highly basic groove of VWF [24] (Figure 7). We postulate that polyP may bind to this highly basic groove in VWF in the absence of FVIII (Figure 7d). Further structural studies are needed to resolve which domain(s) of VWF are specific for binding polyP.

The VWF:RCo assay is the most accepted method for studying the role of VWF in platelet adhesion [15]. Using this method, we discovered that the addition of polyP substantially increases VWF activity in plasmas with depleted FVIII, either with chemically removed FVIII (Figure 3a) or in severe hemophilia patients (Figure 4). Only platelet-size polyP (with a length of more than 45 phosphate residues [1,26]) was effective in the activation of VWF:RCo activity (Supplementary Figure S2). In addition, co-incubating with the enzyme PPX, which specifically degrades polyP, significantly reduced VWF:RCo activity (Figure 3b).

Some evidence indicates that FVIII has greater affinity for VWF than polyP: (i) in normal individuals, in whom levels of circulating FVIII are 40–50 times lower than VWF [27], there is no activating effect of polyP on VWF, and (ii) polyP activates VWF more efficiently in those cases when FVIII levels are extremely low, such as in severe hemophilia A and in type 3 VWD.

The principal complication in replacement therapy for hemophilia A is the generation of autoantibodies (FVIII inhibitors) that occurs in about 30% of hemophilic patients after the infusion of plasma-derived or recombinant FVIII [28]. These autoantibodies inhibit the procoagulant function of FVIII, resulting in increases in morbidity and socio-economic burden [29]. Our results and others suggest that polyP may be used in the design of therapies that replace FVIII to treat hemophilia A. In addition to the activation of VWF in primary hemostasis (this article), polyP improves secondary hemostasis in hemophilia A as well: a previous report showed that polyP reduced the clotting times [30].

The addition of purified FVIII strongly increased the VWF:RCo activity in FVIII-deficient plasmas (Supplementary Figure S2). To our knowledge, this effect has been scarcely studied [31]. Further studies using FVIII-deficient plasmas are urgently needed to give new insights into this mechanism in the regulation of VWF's biological activity.

Finally, research aimed at understanding the physiological consequences of the polyP interactions described here would be of interest. Examining platelet adhesion under flow (in microfluidic devices, for example, as in [32]), shear-induced platelet aggregation [33,34], and in vivo hemostasis models (perhaps using hemophilia A mice) [35], will help to reveal the importance of these interactions in its biological environments.

## 4. Materials and Methods

### 4.1. Plasma Samples

Plasma samples from patients diagnosed with type 1 VWD, were obtained from the Hematology Service of the “Puerta del Mar” Hospital (Cadiz, Spain) as described [13]. Samples from patients diagnosed with hemophilia A (moderate and severe) and type 2 and 3 VWD were obtained from the Hematology Service of the “Virgen del Rocío” Hospital (Seville, Spain) as described [36,37]. Plasma and platelets were separated from blood samples by centrifugation. Approval for this study was obtained from the institutional review board (Ethics Committee). Informed consent was provided according to the Declaration of Helsinki. Plasma chemically depleted in FVIII and isolated VWFs (factor VIII-free and co-purified with factor VIII) were obtained from Haematologic Technologies Inc. (VT, USA). Recombinant human purified factor VIII was obtained from Prospec-Tany (Rehovot, Israel).

### 4.2. Exopolyphosphatase Isolation and PolyP Measurements

Purified recombinant exopolyphosphatase (PPX), from *Saccharomyces cerevisiae*, was obtained as described before [1]. PPX is a highly specific and active enzyme against polyP [38]. Commercial polyP_65_, polyP_45_, and polyP_25_ (Sigma Chemicals Co., St. Louis, MO, USA) were previously isolated via gel filtration with Sephadex G25 to remove orthophosphate, pyrophosphate, and lower-chain polyPs [39]. Exact polyP content was determined from the amount of Pi released upon treatment with an excess of PPX [1]. For all experiments described here, polyP concentrations are expressed in terms of their phosphate residues (Pi).

### 4.3. VWF Ristocetin Co-factor (VWF:RCo) Activity

VWF ristocetin co-factor (VWF:RCo) activity was determined with the Ristocetin Co-factor Assay Kit (Helena Laboratories; Beaumont, TX, USA) and an optical platelet aggregometer (AggRam; Helena Biosciences; or Chronolog Lumi Aggro-meter model 400; Chronolog Corporation).

### 4.4. Surface Plasmon Resonance

The binding kinetics were measured using a XPR36 ProteOn system (BioRad, Munich, Germany) as described before [13]. Briefly, isolated VWF (factor VIII-free or co-purified with factor FVIII, 0.125 mg/mL) was immobilized on a GLM Sensor chip (BioRad). Interaction analysis experiments were performed at a flow rate of 30 μL/min and 1 min contact time at 30 °C. Different concentrations of isolated polyphosphate of 65 residues (polyP_65_, 0.25–13.64 µM) were injected. Immobilization of isolated VWF on sensor chips and association and dissociation with interacting polyphosphate were followed in real-time by monitoring the change in plasmon resonance signal, expressed in resonance units (RU). PolyP concentrations injected were related to the amounts of isolated VWF that can be immobilized on the sensor chip. In a previous study, we used similar micromolar concentrations of polyP [13]. Sensorgrams were analyzed using the ProteOn Manager software. Kinetic constants were obtained using the Langmuir analysis model.

### 4.5. Separation of PolyP by Urea–Polyacrylamide Gel Electrophoresis

Aliquots of 0.46 µg (or indicated amounts) of purified VWF were incubated with 0.8 mM of isolated polyP_65_ for 30 min at 37 °C. Then, samples were separated by 6% urea–polyacrylamide gel electrophoresis, as described [1,13]. Gels were stained with toluidine blue (0.05% toluidine blue, 25% methanol, and 5% glycerol) or with DAPI-negative staining [40].

## 5. Conclusions

Our experiments indicated that polyphosphate (polyP) increased the interaction of von Willebrand factor (VWF) with platelets in plasmas from severe hemophilia A and von Willebrand disease. These plasma samples were scarce in FVIII. As VWF is a key partner for circulating factor VIII (FVIII), we tested the effect of polyP on the VWF–FVIII complex. Various analytical methods revealed that polyP interacts in vitro with VWF only in the absence of FVIII. Interestingly, FVIII has a similar WVF-activating effect as polyP, suggesting that polyP and FVIII could compete in binding to VWF. It would be interesting for future studies to explore the physiological consequences of the reported interactions here.

## Data Availability

Not applicable.

## Supplementary Materials

The following supporting information can be downloaded at: https://www.mdpi.com/article/10.3390/ijms232214118/s1.
