# Supplementary material for: Polyphosphate Activates von Willebrand Factor Interaction with Glycoprotein Ib in the Absence of Factor VIII In Vitro
Source: Int J Mol Sci. 2022 Nov 15;23(22):14118. doi: 10.3390/ijms232214118 (PMC9692336; doi:10.3390/ijms232214118)
Supplement: Supplementary file 1 [file ijms-23-14118-s001.zip › ijms-2004916-supplementary.pdf]

Supplementary Materials
Montilla et al., 2022

Figure S1.

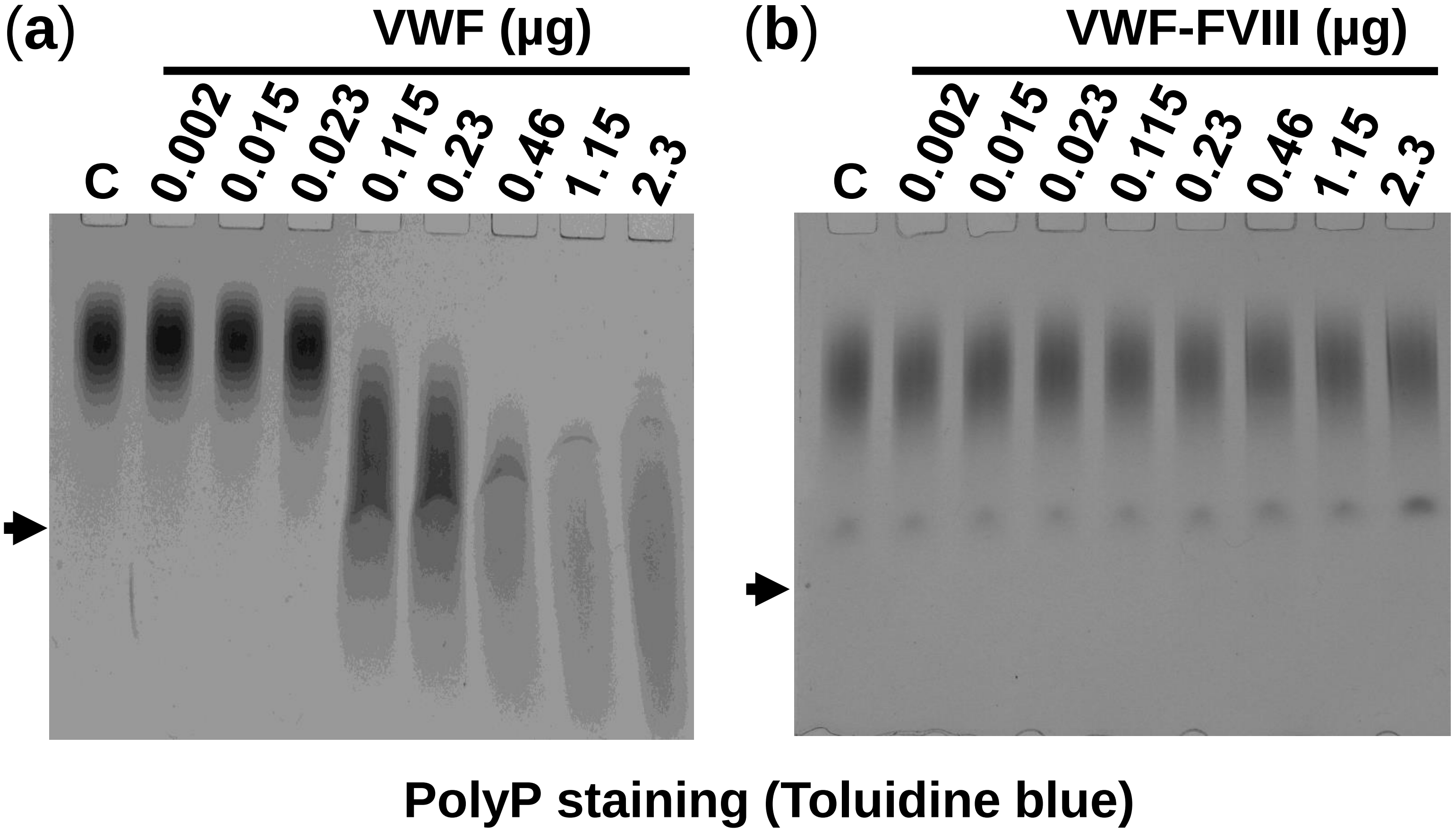

Figure S1. Urea-polyacrylamide gel electrophoresis of polyphosphate and von Willebrand Factor, in the absence or presence of factor VIII. Increasing amounts of von Willebrand Factor (Factor VIII-free) (**a**), or von Willebrand Factor co-purified with factor VIII (**b**), were incubated with 0.8 mM of isolated polyphosphate (polyP$_{65}$) as described in Methods. Samples were loaded onto urea-polyacrylamide gels, separated by electrophoresis, and stained using polyphosphate-specific staining (toluidine blue). Arrows show the mobility of von Willebrand factor on the gels. Representative experiments are shown (n=3).

Supplementary Materials
Montilla et al., 2022

Figure S2.

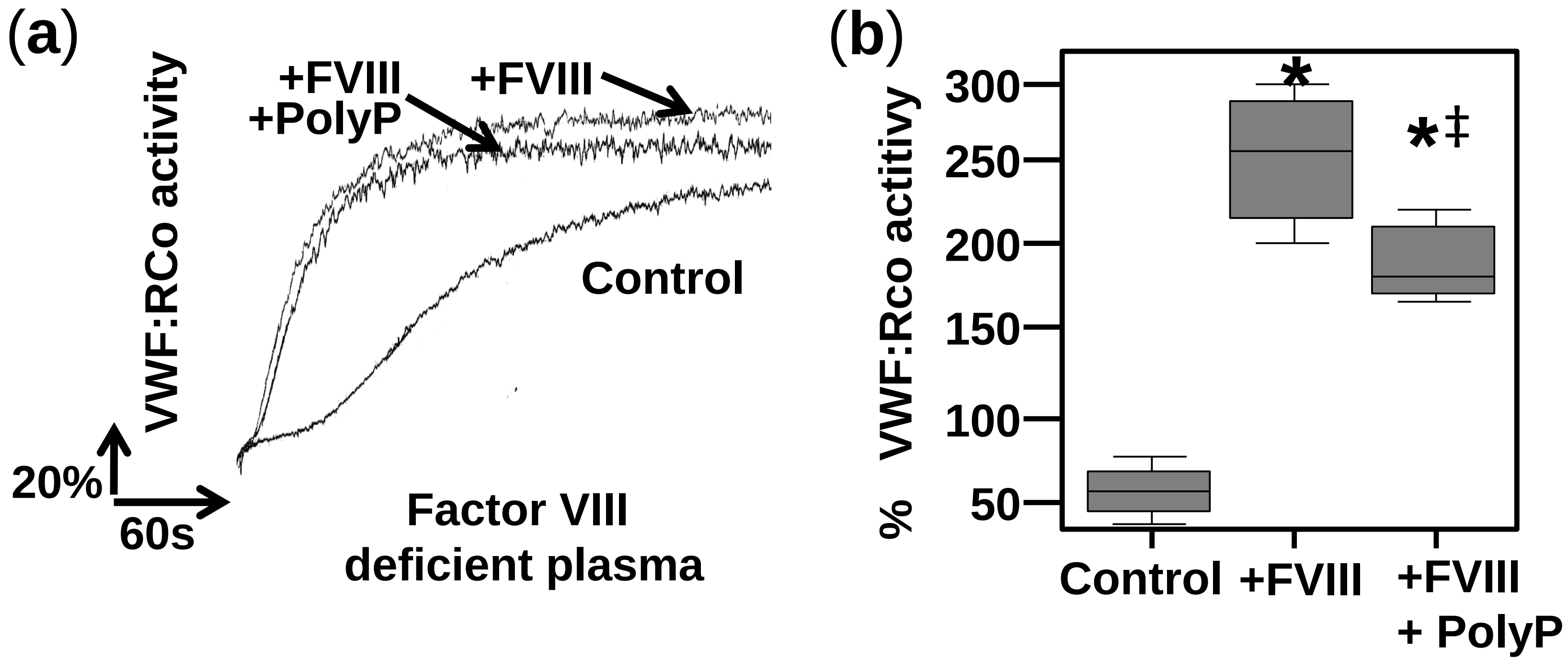

Figure S2. PolyP competes with factor VIII in the activation of von Willebrand Factor ristocetin cofactor activity. (**a**) Aggregation curves of fixed platelets in a ristocetin cofactor assay using factor VIII-deficient plasmas. Prior to the assay, 50 µl of plasmas (diluted 1:2) were incubated with isolated Factor VIII (+Factor VIII), or Factor VIII plus polyphosphate (+Factor VIII +polyP) (0.65 µg Factor VIII and 2.5 µM polyP$_{65}$ were used). Control samples were assayed without Factor VIII or polyP$_{65}$ addition. Representative experiments are shown (n=3). (**b**) Quantification of von Willebrand Factor ristocetin cofactor activity of the result shown in panel (**a**) in three different plasmas. Results are presented in a box-and-whiskers plot. Asterisk indicates a statistical difference of p<.01, determined by Mann-Whitney test, compared to the "Control" measurements. Symbol "‡" indicates a statistical difference of p<.01, determined by Mann-Whitney test, when Factor VIII is compared with Factor VIII plus polyphosphate. Measurements were performed using an automated aggregometer (Helena AggRAM).

Supplementary Materials
Montilla et al., 2022

Figure S3.

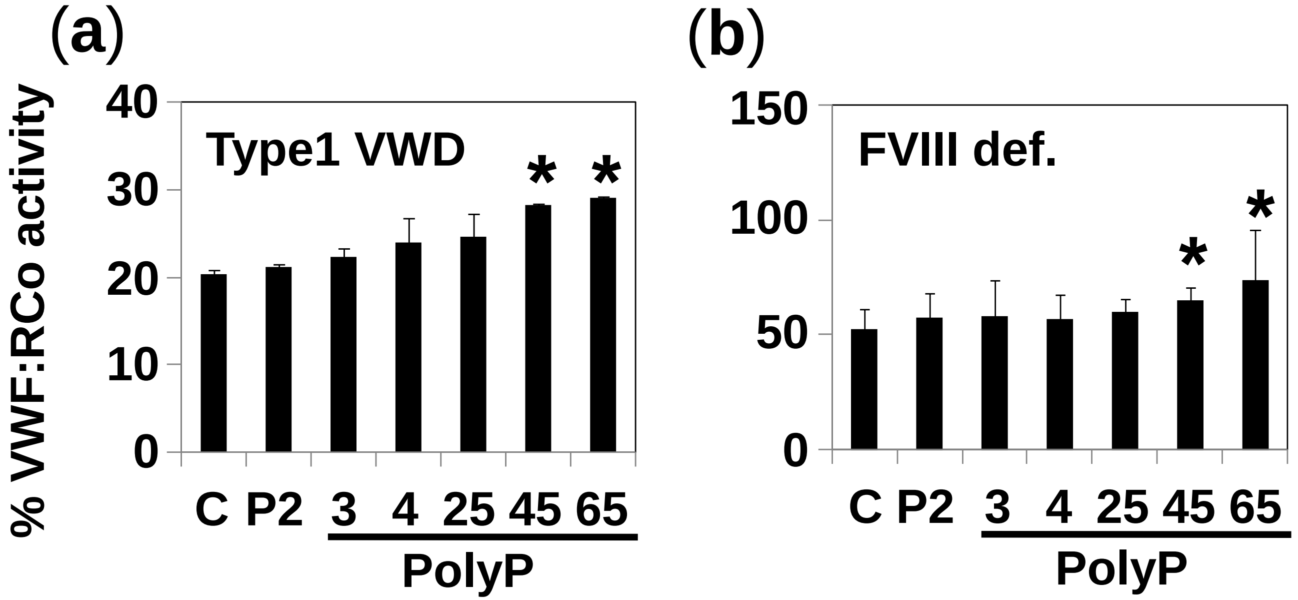

Figure S3. PolyP size determines the activation of von Willebrand Factor ristocetin cofactor activity. A ristocetin cofactor assay was performed using plasma from patients with (**a**) von Willebrand disease type 1, or (**b**) factor VIII-deficient plasmas, in the absence (control, "C") or in presence of 2.5 μM of pyrophosphate (P2), tripolyphosphate (PolyP$_3$), tetrapolyphosphate (PolyP$_4$), PolyP$_{25}$, PolyP$_{45}$, or PolyP$_{65}$. Results are expressed in percentages. Values represent the mean ±S.E from three separate experiments. PolyP and PPi concentrations are expressed in terms of phosphate residues.
